# Supplementary material for: Strongly enhanced THz generation enabled by a graphene hot-carrier fast lane
Source: Nat Commun. 2022 Oct 27;13:6404. doi: 10.1038/s41467-022-34170-3 (PMC9613981; doi:10.1038/s41467-022-34170-3)
Supplement: Supplementary file 1 — Supplementary Information [file 41467_2022_34170_MOESM1_ESM.pdf]

Supplementary Information

I. Device fabrication

The major device fabrication process is discussed in the article. The silicon was first damaged by ion implantation with O+ ions at 100 keV with a flux of 10^{15} cm^{-2}, resulting in a defect density of 0.03 average displacement-per-atom, as verified by a previous work with identical sample preparation conditions[1]. For the pure graphene emitter control group described in the main manuscript, we etched away the silicon layer before the graphene transfer. Another control group with lightly-implanted silicon experiences additional implantation in the silicon Auston switch region. This ensures that the time resolution of the THz field measurement remains identical to the standard device.

For additional gate dependence tests, we fabricated a graphene top gate upon the aforementioned structure. We first PVD 2 nm aluminum on the whole chip. The thin aluminum film is naturally oxidized in the air for 24 hours and forms a buffer layer for Al_2O_3 deposition. We use atomic layer deposition (ALD) to grow a 40-nm-thick Al_2O_3 layer on top of the device. Then a layer of graphene is transferred and patterned as a transparent top gate. Additional window openings and contact metal depositions are also performed for wiring out the relevant contacts.

II. On-Chip Pump Probe Measurements and Scanning Photocurrent Spectroscopy

Supplementary Fig. 1 shows the optical setup for on-chip pump-probe microscopy. The pump beam illuminates the THz emitter. The THz field is coupled to a double metal stripe waveguide. After 100 μm propagation, the signal reaches the Auston switch. Under pulsed illumination, it turns on at an FWHM of 0.7 ps, as shown in Supplementary Fig. 2b, allowing the THz field to be extracted from the waveguide. We can tune the beams' time-delay via the motorized delay line at a dynamic range of 160 ps. The measured current is a convolution of the THz field and Auston switch’s temporal response.

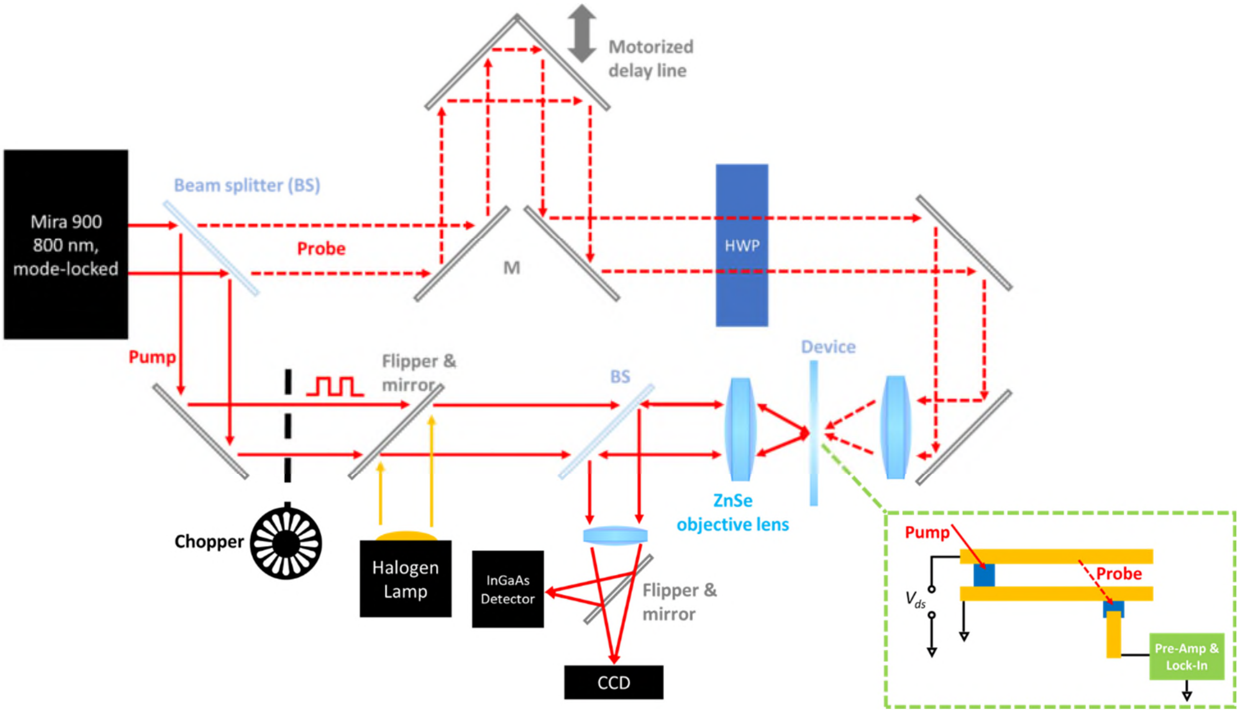

Supplementary Figure 1. The optical setup for on-chip pump-probe spectroscopy. Bottom-right: the device structure and operation condition on chip.

The chopper is placed at the path of the pump beam because the graphene emitter is highly conductive. Placing it on the probe beam would induce a large background signal in the pre-amplifier.

We applied the pump and probe beams from two sides of a transparent silicon-on-sapphire substrate instead of a simpler single-sided setup. This is due to several reasons: For the substrate choice, we need to minimize the THz absorption by the substrate and need thin-layer undoped silicon for fabrication of the Auston switch. Hence the silicon-on-sapphire substrate is chosen. It is transparent and made it possible to pump and probe from two sides of the device. Secondly, the sample is studied later with both temporally and spatially-resolved measurements by integrating the scanning photocurrent spectroscopy into the setup. To modify the position of only the pump beam while maintaining a decent beam quality, we applied motorized stages to control the objective lens position. Hence the objective lens of the two beams must be decoupled, making double-sided illumination the best choice.

We chop the pump beam instead of the probe beam during all measurements. The graphene layer has a significant dark current. Modulating the probe beam would create an RCL-induced background signal. The signal can be substantial and saturate the pre-amplifier. Instead, chopping the pump beam would not cause such a problem. Additionally, a half-wave plate is inserted to ensure orthogonal polarization of the two beams. This prevents interference patterns from creating additional fringes in the measured result.

For scanning THz field generation and photocurrent spectroscopy, a 2D motorized stage moves the objective lens for the pump beam. The beam is hence moved along the device channel. The beam size is estimated to be 1.4 μm, which sets the spatial resolution limit in our position-dependence study. A LabVIEW program in the PC synchronizes the measured signal with the position of the stage.

Gaussian Fittings

The generated THz fields' evolution in time can be conveniently fitted with two halves of Gaussians, one fitting the rise slope and the other fitting the fall slope. The τ values of the Gaussians become a good measure of the rise and fall time of the pulse:

$$f(x) = \frac{1}{\sqrt{2\pi}\tau} e^{-\frac{1}{2}\left(\frac{t-t_0}{\tau}\right)^2} \qquad \tau = \tau_{rise}\ for\ t < t_0, \qquad \tau = \tau_{fall}\ for\ t > t_0$$

Supplementary Fig. 2d shows the fitting of an example amplitude with the two halves of Gaussians.

III. Additional Data:

THz Generation

Supplementary Fig. 2 shows additional data of on-chip pump probe measurements. We scanned the pump beam across the channel of the emitter (along white dotted line in Supplementary Fig. 2a inset) to study the spatial dependence of the THz excitation. The THz generation happens across the whole graphene channel. It is strongest near the left contact, which was also observed and discussed in previous

literatures[2,3,4]. The extracted data in supplementary Fig. 2b indicate a linear dependence of field amplitude on the channel bias in the graphene-on-silicon devices.

The lifetime of transient carriers in silicon Auston switch is characterized with the same on-chip pump-probe measurement with identical layout to our main measurements, with the only difference that the emitter does not have a graphene layer on top. Supplementary Fig. 2c shows the pulse generated by the simple silicon emitter. The observed pulse has a FWHM of 0.9 ps, corresponding to a carrier lifetime of 0.65 ps by deconvolving the emitter and detector transients assuming exponential decays. The value matches well with the value (0.6 ps) observed in samples prepared with identical conditions in previous literatures[5,6].

Signal from Pure Graphene Device

Our pure graphene device (Fig. 3a) behaves differently from the previously reported graphene THz emitters[7,8]. There is very weak channel bias dependence of the pulse amplitudes, which suggests that edge effects are dominating the THz generation, contrary to both the previous observations of a strong photoconductive effect and our hybrid PCS. We attribute the discrepancy to the test condition difference between the experiments. In our test, the pump beam is focused on the edge, so that the contribution from a single edge dominates over the contribution from a very limited area of channel. In the other works, a larger beam spot illuminates the whole channel, which may generate more signal from the photoconductive effect.

Photocurrent Measurements

We also examine the photocurrent extracted from the device. Unlike the emitted THz field, the photocurrent has a nonlinear dependence on the channel bias (Supplementary Fig. 3a). Additional

spatially-resolved measurement (Supplementary Fig. 3d,e,f) also suggests that the photocurrent collection is most efficient at the edge, which is different from the THz field results. Photocurrent autocorrelation (Supplementary Fig. 3b) suggests intrinsic carrier dynamics with full-width-half-magnitude (FWHM) of 0.3 ps, which is even faster than the THz generation observations. More interestingly, the photocurrent harvested is almost three orders of magnitude higher with graphene than the case without graphene.

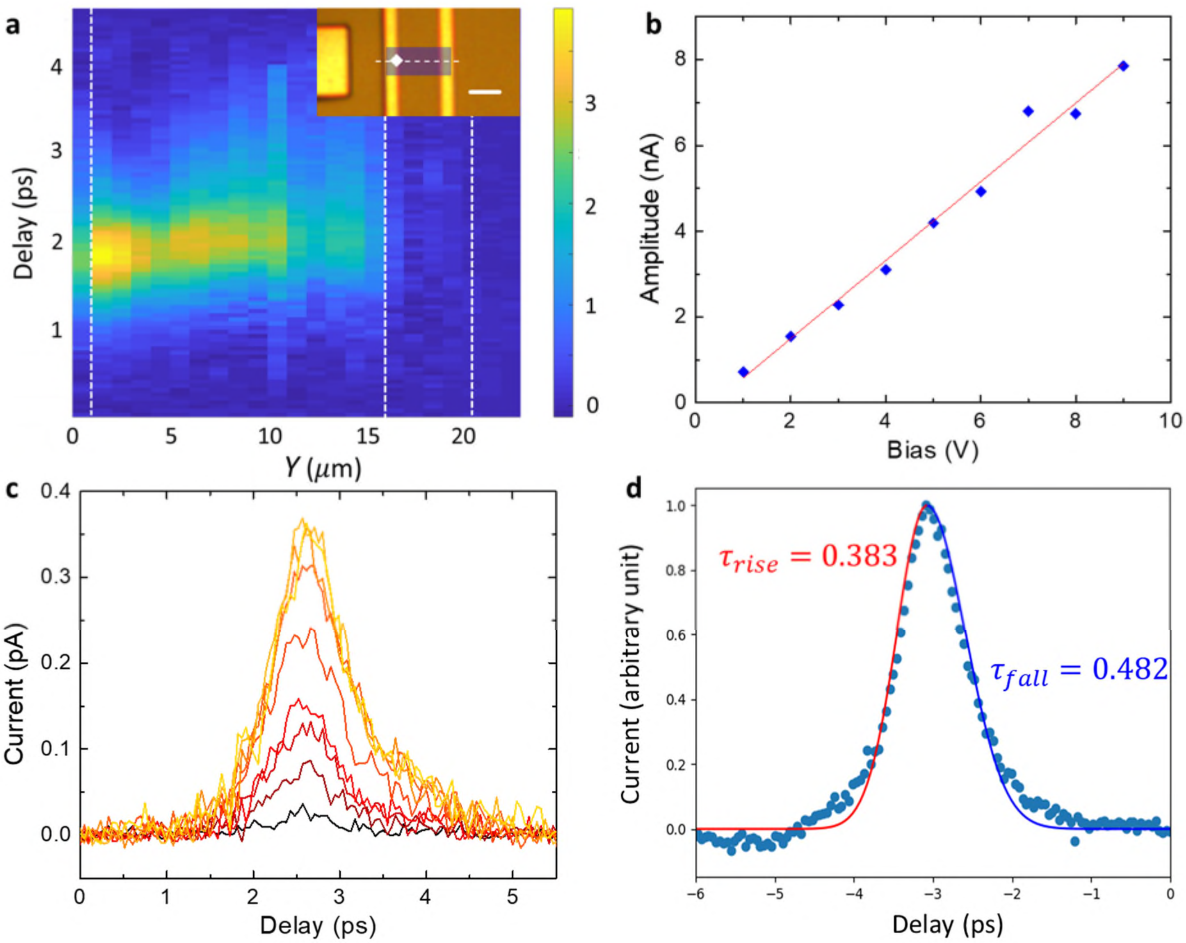

Supplementary Figure 2. **a** Field amplitude plotted in color with dependence on both time and pump beam position. Inset: Emitter image under optical microscope. The graphene channel is rendered with enhanced color. Pump beam scans along the dotted line. The white diamond marks the pump position in all other tests with fixed pump beam. Scale bar: 10 μm. **b** Peak field amplitude at different channel bias in Fig. 2a. The field amplitude is linearly dependent on the channel bias. Red line: fitting with lease square

linear regression. **c** Pump probe measurements of THz generation of a simple silicon Auston switch, captured by another silicon Auston switch triggered with the probe beam. Black to yellow: channel bias = 1 V to 9 V, with 1 V stepped increase. The measurement sets the temporal resolution of our studies. Pump power = 3 mW, probe power = 10 mW. The test condition is consistent with Fig. 2a, so that we can derive the power gain (Fig. 2b) from it. **d** The fitted Gaussians from an example measured field.

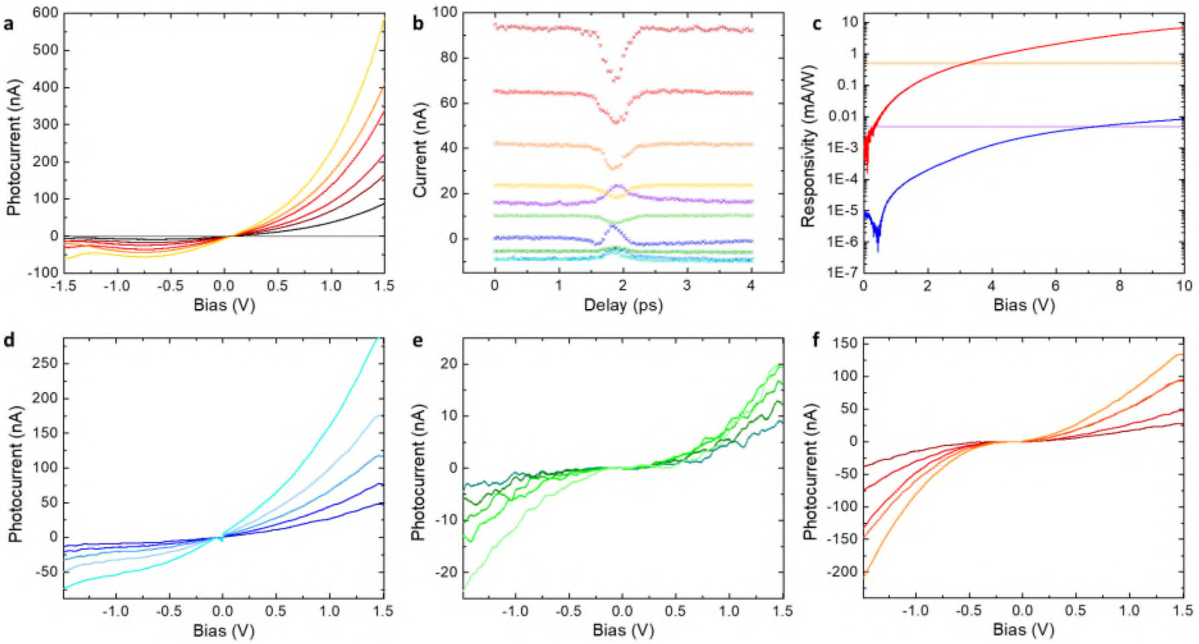

Supplementary Figure 3. **a** Photocurrent from the device under various powers. The nonlinear channel bias dependence may originate from local band alignment change under different bias. Black to yellow: illumination power = 0.1, 0.2, 0.3, 0.5, 0.7, 1.0 mW. **b** Photocurrent autocorrelation with the pump beam (1 mW) and probe beam (0.5 mW) illuminating the same region on the device. Different colors correspond to a 0.2-V stepped bias change across the channel from -1 V (violet) to 1V (crimson). The FWHM of the peak is 0.3 ps for 1V bias. **c** Responsivity comparison of 2D-3D devices (red) and the graphene free counterpart (navy). The insertion of the graphene layer results in about 800 times higher responsivity than the typical Auston switch. Light orange bar: reported responsivity of unbiased exfoliated graphene at metal edges; light violet bar: reported responsivity of unbiased CVD-grown graphene at metal edges contributed by lateral photo-Dember effect. **d** - **f** Spatially-resolved photocurrent measurement with excitation at lower edge **d**, middle of channel **e**, and upper edge **f**. Colors from dark to light correspond to illumination power of 25, 50, 100, 200, and 250 μW respectively. With the beam centered at the upper and lower edge, the photocurrent is an order of magnitude larger than the case with the beam centered in the channel's center. We also observe a stronger nonlinearity with sweeping channel bias at the edges.

IV: Gate Tuning of Band Alignment

As explained in the main context, the silicon layer beneath the device is depleted under negative gate bias. Under positive gate bias, it accumulates electrons. This is further verified by measuring 7 other devices of same structure with the one in Fig. 4a. All the n-branch shows moderate slope compared with the p-branch, as a result of counter-doping effect of the silicon layer.

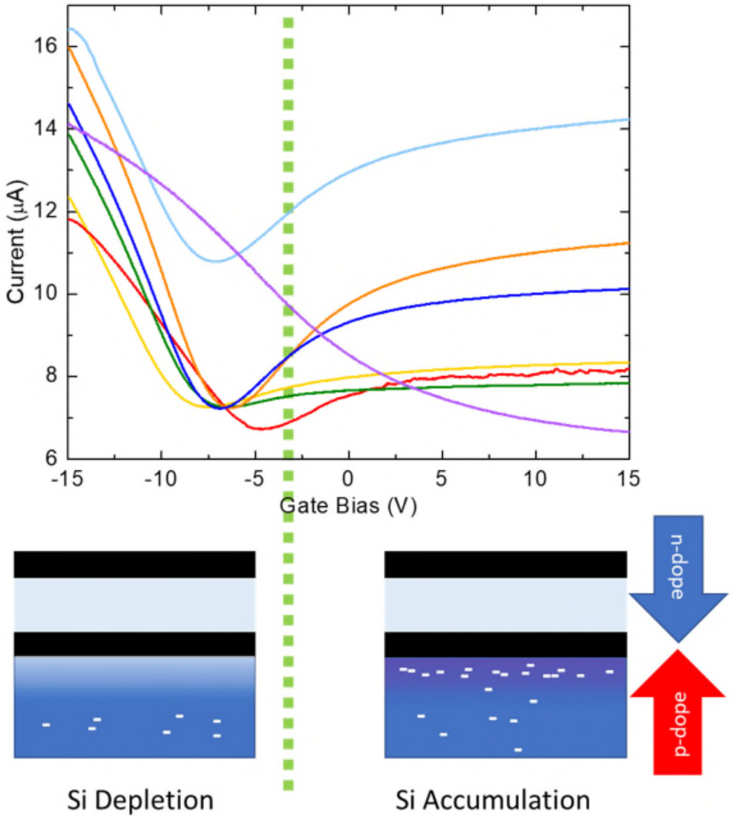

Supplementary Figure 4. Transfer curves of 7 different devices at Vds = 100 mV. All curves show inefficient n-doping of the graphene channel at positive gate bias. The graphene layer only partially screens the electric field, causing an additional gating effect in the silicon layer underneath. The charge accumulation in the silicon layer counter-dopes the graphene layer, resulting in a smaller slope.

To further support the hypothesis, we performed C-V measurements between the two layers of graphene. The measurement was performed at 2 MHz, using the setup shown in Supplementary Fig. 5. The device is biased under a DC gate bias Vg, combined with an AC bias Vsrc. The voltage measured by the oscillator, Vosc, is given by:

$$\frac{V_{osc}}{V_{src}} = \frac{j\omega R_0 C_x}{1 + j\omega R_0 C_0 + j\omega R_0 C_x}$$

The capacitance of the device is estimated in orders of magnitude to be Cx ~ $0.01C_0$ based on the capacitor geometry. Besides, at 2 MHz, we also have $j\omega R_0 C_0 \gg 1$. Hence the equation above can be approximated into:

$$\frac{V_{osc}}{V_{src}} \cong \frac{C_x}{C_0}$$

By comparison of the AC amplitudes of Vosc and Vsrc, the capacitance can be estimated with the gate dependence shown below. The value is consistent with our estimation in orders of magnitude. More importantly, the gate dependence shows an increase of capacitance at high positive bias, which can be explained by the parasitic capacitance between silicon and graphene channel. The capacitance is increased because of the accumulation of electrons, as happens in a typical silicon FET's CV measurement.

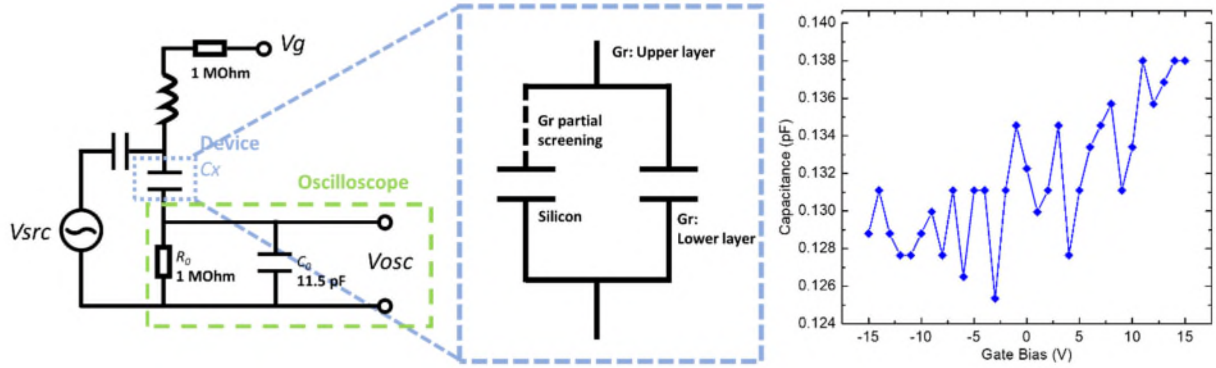

Supplementary Figure 5. Measurement circuitry for gate capacitance extraction. A parasitic capacitance between silicon and graphene channel exists due to partial screening of field through graphene. Right: increase of total capacitance due to DC gating.

---

[1] Lui K P H, Hegmann F A. Fluence-and temperature-dependent studies of carrier dynamics in radiation-damaged silicon-on-sapphire and amorphous silicon[J]. Journal of applied physics, 2003, 93(11): 9012-9018.

[2] Ralph S E, Grischkowsky D. Trap-enhanced electric fields in semi-insulators: The role of electrical and optical carrier injection[J]. Applied physics letters, 1991, 59(16): 1972-1974.

[3] Keil U D, Dykaar D R. Ultrafast pulse generation in photoconductive switches[J]. IEEE Journal of Quantum Electronics, 1996, 32(9): 1664-1671.

[4] Castro-Camus E, Lloyd-Hughes J, Johnston M B. Three-dimensional carrier-dynamics simulation of terahertz emission from photoconductive switches[J]. Physical Review B, 2005, 71(19): 195301.

[5] Ketchen M B, Grischkowsky D, Chen T C, et al. Generation of subpicosecond electrical pulses on coplanar transmission lines[J]. Applied Physics Letters 48(12): 751-753 (1986).

[6] Doany F E, Grischkowsky D, Chi C C. Carrier lifetime versus ion‐implantation dose in silicon on sapphire[J]. Applied Physics Letters 50(8): 460-462 (1987).

[7] Hunter N, Mayorov A S, Wood C D, et al. On-chip picosecond pulse detection and generation using graphene photoconductive switches[J]. Nano letters, 2015, 15(3): 1591-1596.

[8] Tong J, Muthee M, Chen S Y, et al. Antenna enhanced graphene THz emitter and detector[J]. Nano Letters, 2015, 15(8): 5295-5301.
